# Observational real-world analysis of dose-dependent effects of *Mitragyna speciosa (Korth.)* on focus and energy

**DOI:** 10.3389/fphar.2026.1838148

**Published:** 2026-08-28

**Authors:** C. Miyabe Shields, Riley Kirk, Michael Chan, Paloma Lehfeldt, Paula N. Brown

**Affiliations:** 1 Network of Applied Pharmacognosy, Whitefield, NH, United States; 2 Natural Health and Food Products Research Group, Centre for Applied Research and Innovation, BC Institute of Technology, Burnaby, BC, Canada; 3 Scientific Association for Botanical Education and Research, Cleveland, OH, United States

**Keywords:** dose-dependance, energy, focus, kratom (*Mitragyna speciosa* korth.), mitragynine, observational studies, real-world data

## Abstract

**Background:**

*Mitragyna speciosa* is a deciduous evergreen tree native to Southeast Asia. The term “kratom” is commonly used to refer to both the tree and its leaves, which have a long history of use as a restorative to provide energy, focus and relief of minor aches and pains associated with physical exertion. Consumption of kratom leaves, either chewed or prepared as a tea or decoction, continues in Southeast Asia to this day. Similar to Southeast Asia, U.S. consumers report taking kratom to alleviate pain, increase energy, and improve negative mood states. We have evaluated the real-world effectiveness of a full-spectrum proprietary kratom extract at doses aligned with traditional ethnopharmacological use, specifically for effects that match the traditional profile of stimulating and focusing effects.

**Methods:**

This study represents a secondary analysis of data collected during a real-world, longitudinal observational study of adult U.S. participants using a kratom product composed of proprietary full spectrum leaf extract, designed to mimic a traditional kratom tea. Phytochemical equivalence to traditional preparations was established by comparing the alkaloid profile and presence of plant phenylpropanoids. Participants completed daily structured surveys over a 21-day period. Linear mixed-effects models were used to evaluate within-subject changes across baseline, week 1, and week 2 periods.

**Results:**

Chemical analyses confirmed that the presence and relative ratios of alkaloids were comparable to those found in traditional kratom tea, supporting the extract as a standardized representation of traditional decoctions. At the 20 mg mitragynine-equivalent dose, participants showed statistically significant improvements in focus, problem solving, mental clarity, and energy (p < 0.05).

**Conclusion:**

A kratom product formulated to be dose-aligned with a traditional kratom tea serving is associated with stimulant-like benefits, increasing energy and focus. This effects profile implies that the pharmacological activity at this dose likely reflects non-opioid receptor mechanisms.

## 1 Introduction

Kratom, *Mitragyna speciosa* (Korth.) Havil (Rubiaceae), is a tropical deciduous tree native throughout Southeast Asia, particularly in Thailand, Malaysia, Indonesia, Philippines, and Vietnam (Cinosi et al., 2015). The leaves of kratom have been used for centuries as a food and medicine by the indigenous people of these regions (Tanguay, 2011; Raffa, 2015; Brown et al., 2017). Reference to kratom consumption by Native Malayans appears in the literature as early as 1836, and a description of methods of consumption, particularly in the region that is now southern Thailand was reported by Wray in 1907 (Low, 1836; Wray, 1907; Jansen and Prast, 1988). Traditionally, kratom leaves are chewed either fresh or dried, consumed as a decoction or tea made from boiling leaves in water, and used in cooking, e.g., as soup (Wray, 1907; Assanangkornchai et al., 2007; Vicknasingam et al., 2010; Hassan et al., 2013; Brown et al., 2017).

Traditionally, kratom consumption was reported to increase work efficiency by improving physical stamina and alleviating aches and pains associated with physical labor (Watanabe et al., 1997; Babu et al., 2008; Vicknasingam et al., 2010; Adkins et al., 2011; Tanguay, 2011; Ahmad and Aziz, 2012; Barceloux, 2012). Although the consumption of kratom leaves continues in Southeast Asia today as a culturally accepted stimulant, recent surveys also report use to reduce dependence and side effects associated with opioid and methamphetamine use (Vicknasingam et al., 2010; Adkins et al., 2011; Saingam et al., 2013; Warner et al., 2016; Saref et al., 2020; Charoenratana et al., 2021). It is this latter use that has attracted the attention of researchers amidst a global opioid crisis, while also raising concerns about safety.

Kratom’s presence in North America is not well documented (Henningfield et al., 2022); surveyed commercial export data from the Indonesian Kratom Grower’s Association to the U.S. suggests 10–16 million U.S. kratom consumers (Henningfield et al., 2019). This is consistent with a nationally representative online survey that estimated 10.5 million Kratom consumers in 2019 (Covvey et al., 2020). Similar to Southeast Asia, where scientific and ethnographic literature describes kratom consumption as motivated by “beneficial”, “labor sustaining,” “therapeutic,” “mood,” and “well-being” enhancing attributes, the majority of U.S. respondents report that kratom improves health and well-being. Specifically, respondents report using kratom to alleviate pain, increase energy, and improve negative mood states (Grundmann, 2017; Swogger and Walsh, 2018; Garcia-Romeu et al., 2020; Smith et al., 2022a; Grundmann et al., 2023b; Grundmann et al., 2023c; Smith et al., 2024b; Piercey et al., 2025). A large proportion of American consumers also report using kratom as a means to reduce or abstain from over the counter (OTC) and prescription medications for which they found kratom’s safety and side effect profile more tolerable (Smith and Lawson, 2017; Coe et al., 2019; Smith et al., 2021; Smith et al., 2023; Settle et al., 2024).

Although best known for the action of mitragynine, kratom leaf offers an entourage effect of multiple relevant bioactive compounds that expand its properties in the body, including other chemical classes like flavonoids, polyphenolic compounds, triterpenoids, triterpenoid saponins, monoterpenes, and secoiroids (Brown et al., 2017). Beyond mitragynine, which comprises of between 65%–80% of the total alkaloid extract, other relevant alkaloids found in appreciable amounts include paynantheine (8%–10%), speciogynine (6%–8%), and speciociliatine (Todd et al., 2020; Kamble et al., 2022; Sengnon et al., 2023) (0.8%–2%) with the total weight of alkaloids in the leaves equaling 0.5%–3% (Hassan et al., 2013; Kruegel and Grundmann, 2018). Another notable alkaloid often associated with kratom is 7-hydroxymitragynine (7-HMG) (Kamble et al., 2019). Although initially thought to be present in kratom leaf, it is now recognized that 7-HMG is an oxidative byproduct of mitragynine, observed in post-harvest kratom leaf at trace levels (0.003%–0.04% w/w) (Chear et al., 2021; Zhang et al., 2022; Karunakaran et al., 2024). 7-HMG has gained recent attention as products have emerged that contain very high levels of 7-HMG, achievable only by synthetic means. Concentrated 7-HMG products are becoming increasingly present in the U.S. marketplace and in contrast to traditional kratom preparations have been reported to have a notably different dose-effects profile (Alsbrook et al., 2025). These products are often mislabeled as kratom, may contain other unidentified constituents not found in kratom leaf and pose a significant risk to public health (Smith et al., 2024a; Brown et al., 2026; Gour et al., 2025).

Traditional servings of kratom leaf as teas or decoctions have shown promise as a substitute or alternative for stimulant beverages like coffee. This is likely due to the alkaloids acting poly-pharmacologically on multiple key receptors in the body. From Southeast Asia low to moderate doses of 1–5 g of leaves are reported for stimulant effects (Prozialeck et al., 2012; Grundmann, 2017; Smith et al., 2022b). In contrast, higher doses have been reported to have sedative effects (Prozialeck et al., 2012; Grundmann, 2017; Smith et al., 2022b). Recent studies have lent support to this dose-effect relationship; a human pharmacokinetic study found a single dose of 2 g of kratom leaf powder (∼20–26 mg of mitragynine equivalent) produced measurable plasma levels without significant sedation or opioid-like effects, implying a different mechanism of action (Huestis et al., 2024). Despite the primary traditional use of kratom as a restorative, there still remains limited clinical data or evidence of kratom’s cognitive enhancement properties in the research literature (Prevete et al., 2025; Smith et al., 2024b; Rogers et al., 2024).

Pharmacological studies of kratom primarily focused on its alkaloids and in particular the predominant alkaloid mitragynine, provide some insights into the dose response effects (Hiranita et al., 2019; Kamble et al., 2020; Obeng et al., 2020; Obeng et al., 2022). *In vitro* and *in vivo* studies suggest that at lower concentrations, mitragynine exhibits partial agonist activity at α2-adrenergic receptors (Ki = 2.3–4.4 µM), as well as interactions with dopaminergic and serotonergic systems. These activities may be responsible for the stimulant-like effects of “alertness”, “motivation”, and “cognitive clarity” (Matsumoto et al., 1996; Hazim et al., 2014; Obeng et al., 2020). An extract of kratom showed affinity to dopamine D1 receptors (Ki = 3.38 µM) in *ex vivo* binding studies (Stolt et al., 2014), while paynantheine and speciogynine, exhibited high affinity at serotonin 5-HT1A receptors, which may contribute to anxiolytic and mood-regulating mechanisms of chemically diverse kratom formulations (León et al., 2021).

These mechanisms contrast with those observed at higher doses, where mitragynine acts as a partial agonist at the μ-opioid receptor and a competitive agonist at the δ- and κ-opioid receptors (Kruegel et al., 2016; Váradi et al., 2016; Obeng et al., 2020), exerting sedative and analgesic effects. Functional assays revealed that mitragynine shows G-protein biased agonism with no measurable recruitment of β-arrestin 2, a property linked to a reduced risk of respiratory depression and other classical opioid-related adverse effects (Gutridge et al., 2020; Todd et al., 2020). Rodent studies further demonstrated that at higher doses (15–30 mg/kg), mitragynine reduces inflammatory signaling via downregulation of COX-1, COX-2, and TRPV1, contributing to its analgesic and anti-inflammatory activity (Mat et al., 2023).

Collectively these findings indicate that kratom’s pharmacological profile may shift with dose; with low doses preferentially engaging in non-opioid pathways associated with psychostimulant-like effect, and higher doses associated with the opioid receptor activity of analgesia and sedation. This dose-dependent receptor engagement may explain the divergent subjective effects of kratom across the dosage spectrum. Notably, the stimulating properties of kratom differ from those of traditional caffeine-containing stimulants such as coffee and tea, as they appear to arise from modulation of adrenergic and dopaminergic receptor systems rather than central adenosine receptor antagonism, potentially resulting in a more sustained sense of alertness without the jitteriness or abrupt energy crashes commonly associated with caffeine (Higgins et al., 2018; Yusoff et al., 2022; Obeng et al., 2024; Dobrek, 2025).

Beyond the substantial therapeutic potential attributed to kratom and its constituent alkaloids, adverse events have increasingly been reported among consumers in the U.S. (Prozialeck et al., 2019; Li et al., 2023). These incidents are often associated with non-traditional or commercially modified preparations in which alkaloid concentrations greatly exceed those found in traditional ethnopharmacological contexts (Obeng et al., 2020; Todd et al., 2020; Hanapi et al., 2021). In particular, enrichment or synthetic augmentation of 7-hydroxymitragynine, a potent oxidation product of mitragynine, has been identified in some products erroneously labelled as kratom (Lydecker et al., 2016; Sharma and McCurdy, 2021; Brown et al., 2026; Hill et al., 2025). Such alterations in alkaloid composition and potency may underlie the higher incidence of toxicity reported in these modern formulations (Obeng et al., 2020; Hanapi et al., 2021). Similar to the benefits of kratom, adverse effects are dose and duration dependent with individuals ingesting up to 5 g of kratom leaf unlikely to present negative effects (Singh et al., 2014; Grundmann, 2017; Garcia-Romeu et al., 2020).

Focusing on ethnopharmacologically relevant doses can serve to increase our understanding of the potential benefits of this medicinal plant within the context of its traditional use. Traditional kratom tea (TKT) is typically prepared as decoction by simmering leaves (fresh or dried) for a period of time ranging from minutes to hours (Wray, 1907; Ahmad and Aziz, 2012; Saingam et al., 2013; Singh et al., 2025). The kratom product (KP) described in this analysis was developed to mimic TKT. Delivering 20 mg of mitragynine per serving, the KP falls just below the reported range for single traditional serving (21.0–79.0 mg of mitragynine) and within the range of the reported minimal effective threshold of 5.9–164.9 mg mitragynine (Vicknasingam et al., 2010; Singh et al., 2019b; Leong Abdullah et al., 2021; Grundmann et al., 2022). This serving size is also consistent with recent cross-sectional observational studies in Malaysia, where traditionally prepared decoctions were consistently found to contain 21–33 mg mitragynine per serving, with daily consumption of 72–115 mg mitragynine (Singh et al., 2019b).

The present analysis reports the effects of KP from a longitudinal, real-world observational study, exploring patterns of use and subjective outcomes rather than to establish clinical efficacy or causality. As an observational, non-interventional investigation, the findings should be interpreted as exploratory and reflective of real-world use of KP in a non-traditional setting. While many kratom products currently available in the North American market are highly concentrated extracts containing only the alkaloid fraction from the kratom leaf, the KP in this study reflects the phytochemistry present in kratom leaf and traditional preparations from kratom leaf.

## 2 Materials and methods

### 2.1 Description of materials, KP and TKT

KP is a commercially available liquid kratom product comprised of a full spectrum kratom leaf extract that was independently verified to contain 5.0% ± 0.5% (w/w) mitragynine diluted in water to deliver a standardized serving of 20.0 ± 0.4 mg of mitragynine equivalent per serving. To compare KP with TKT, 5 lots of KP was obtained from the manufacturer and 5 batches of TKT were prepared analogous to how traditionally ingested kratom teas are brewed in Southeast Asia (Ahmad and Aziz, 2012; Singh et al., 2025; Wray, 1907; Saingam et al., 2013). Each batch of TKT was prepared using crushed *M. speciosa* (Korth.) Havil leaves obtained from Kalimantan, Indonesia and prepared using a tea preparation method described by Singh, Lowe, and Berthold, 2025. Briefly, dried kratom leaves (5–10 g) were added to water (100–500 mL), heated to 70 °C–90 °C and left for approximately 1 h after which the liquid was filtered to remove the solid leaf materials. TKTs were allowed to cool to room temperature prior to analysis.

The multiple batches (n = 5) of KP and TKT were analyzed using the validated Official Method of Analysis AOAC 2017.14 to compare the relative abundance of mitragynine and the major alkaloids (Mudge and Brown 2017). To demonstrate that the kratom leaf extract used to formulate the KP was a full spectrum leaf extract, four phenylpropanoids (chlorogenic acid, rutin, hyperoside and quercetin) were measured by a previously developed high-performance liquid chromatography method (Mudge et al., 2016).

### 2.2 Study design and data collection

This real-world observational study is a secondary analysis with non-random sampling through a third-party health and wellness data software company (MoreBetter Ltd., Hyattsville, United States) who provided anonymized data to the investigators under a data use agreement. For the self-reported focus and energy effects, participants served as their own controls, with each participant’s baseline measurements used as a comparison to the observational period. This study has been determined to be exempt from IRB review under category 4ii, as detailed in 45 CFR 46.104(d) (BRANY IRB #25-12-503-2301, Determination #262287).

Participants were recruited through social media platforms (Instagram, TikTok), email listservs targeting health and wellness audiences, and podcast advertisements. Recruitment materials provided a brief study overview and a link to the enrollment survey for eligibility screening - adults over the age of 21, US residents, with internet access, not pregnant or trying to become pregnant, and without any major health indications. Eligible participants completed an enrollment survey, including an electronic informed consent for participation, and began a 7-day baseline period with no product use, followed by 2 weeks of observational reporting of KP consumption followed by structured self-reported questionnaires. Each evening, participants received a survey prompt via SMS text message and email and submitted responses via a structured questionnaire. Participants were not assigned to a dose group; KP dosage and frequency were self-selected and self-reported at each use, consistent with real-world use patterns; differential dosages were categorized *post hoc* for further analyses by individual daily data session responses. If participants reported they did not consume the product that day, no further questions were asked. Data was stored on secure servers and anonymized prior to this analysis.

### 2.3 Measures

Self-reported focus and energy levels were assessed daily using a structured questionnaire with each question rated on a 5-point Likert scale. The baseline metrics for each participant were calculated as the average of the 7 days prior to KP use initiation. After the baseline period, participants began daily reporting regardless of KP use. Participants reporting KP use were asked the dosage and frequency of KP use as well as any perceived positive and negative effects. When participants did not report KP use, no follow-up questions were asked. The focus and energy level assessment questions were included throughout the entire study.

### 2.4 Statistical analyses

The full dataset contained data from 82 participants with varying levels of completion. The datafile was filtered for completion of the full 21 days of data collection (*n* = 35) for our analysis. The observational data were divided into three phases: baseline (days 0–7, prior to KP use initiation), week 1 (days 1–7 of observed KP use), and week 2 (days 8–14 of observed KP use). During week 1 and week 2, data was filtered to only include responses during KP use days and grouped by reported dose range (20 mg, 21–40 mg mitragynine equivalent). For each participant, responses to the survey questions were averaged across all available days within the given phase to produce a baseline mean, and two observational means (week 1 and week 2) for each question. Each participant’s average score was calculated based on the number of days each individual reported consuming KP, with each participant having multiple scores contributing to the averaged score used for comparison in this analysis.

Comparisons of the two observational participant mean values to the baseline participant mean values were conducted using linear mixed-effects models (LMMs) to account for repeated measures within individuals. The models included time period (baseline, week 1, week 2) as a fixed variable and participant ID as a random intercept to control for within-subject correlations. Separate analyses were conducted for each outcome variable. Statistical significance was set at p < 0.05. Analyses were performed using Python 3.12 (Anaconda distribution) with pandas, numpy, and matplotlib packages for data management and visualization, and the statsmodels package for mixed-effects modeling. Violin plots of response distributions were generated using the seaborn package.

## 3 Results

### 3.1 Product characterization

**FIGURE 1:**
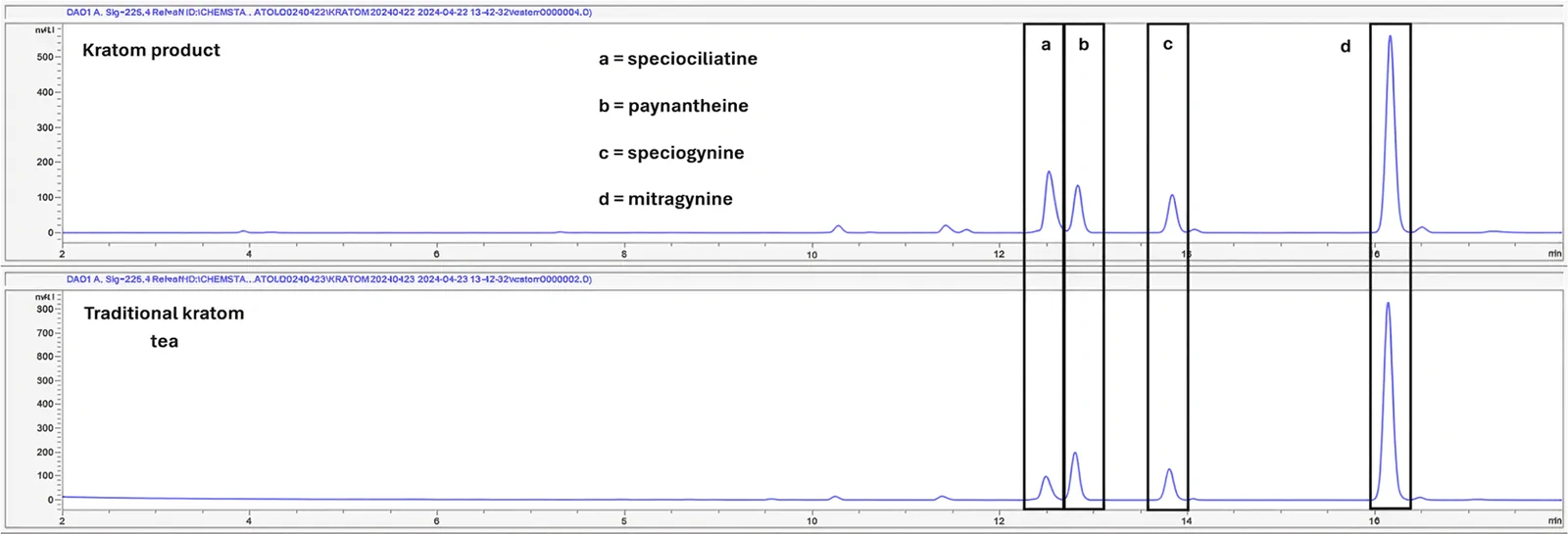
LC chromatograms of the KP (top; red) and TKT prepared using crushed kratom leaves from Kalimantan, Indonesia according to tea preparation methods described by Singh et al. (2025) (bottom; blue) showing profile of major alkaloids.

To demonstrate phytochemical equivalence, the KP was chemically compared to a TKT. The relative composition of mitragynine, speciociliatine, paynantheine and speciogynine were calculated using mitragynine as a surrogate reference calibrant for all identified major alkaloids (Table 1) for 5 lots of KP and 5 batches of TKT. The chromatographic fingerprint of TKT and KP is shown in Figure 1. The pattern is consistent showing the major identified peaks, speciociliatine, paynantheine, speciogynine and mitragynine. The results demonstrate that the presence and compositional ratios of the major alkaloids are conserved from TKT in the KP.

**TABLE 1:** Alkaloid levels expressed as % relative abundance of five lots of the KP and TKT.

​	Relative % of total alkaloids			
Sample lot	Mitragynine	Speciociliatine	Paynantheine	Speciogynine
TKT (n = 5)	63.3	14.2	13.1	9.4
	62.5	11.4	15.9	10.2
	55.5	13.6	17.0	13.9
	66.9	10.7	12.3	10.1
	53.3	14.8	17.8	14.1
Mean	60.3	12.9	15.2	11.5
Standard error	2.5	0.8	1.1	1.0
KP (n = 5)	65.2	14.3	12.4	8.4
	64.3	14.9	12.8	8.0
	57.6	11.6	16.9	13.9
	65.2	14.3	12.4	8.1
	56.4	12.8	15.6	15.2
Mean	61.7	13.6	14.0	10.7
Standard error	2.0	0.6	0.9	1.6

Analysis of the phenylpropanoids in kratom leaf material, KP and TKT demonstrated that all four targeted marker compounds (chlorogenic acid, rutin, hyperoside and quercetin) were present. As shown in Table 2, rutin and quercetin content are reasonably consistent in the leaves, TKT and KP. There was an observed increase in the relative amounts of hyperoside and decrease in chlorogenic acid when comparing KP with TKT and kratom leaves, likely due to variance in solubility of these specific compounds in the extracting solution. While the manufacture of the kratom extract used to formulate the KP may selectively extract hyperoside over chlorogenic acid, this effect would be limited by the 5:1 dry extract ratio. In other words, any extracted constituent in a 0.5 g serving of the extract can be no greater than the amount of the same constituent in 2.5 g (0.5 g × 5) of dried kratom leaf raw material.

**TABLE 2:** Relative abundance of 4 phenylpropanoid marker compounds in kratom leaf. TKT and the KP.

Phenylpropanoid marker	Leaf (n = 3)	TKT (n = 5)	KP (n = 5)
Chlorogenic	22.1% ± 4%	17.1% ± 5%	10.2% ± 4%
Rutin	14.3% ± 4%	17,9% ± 5%	14.6% ± 2%
Hyperoside	7.8 %± 3%	11.6% ± 5%	18.0% ± 2%
Quercetin	55.8% ± 5%	53.0% ± 10%	57.2% ± 5%

Overall, the alkaloid and phenylpropanoid data collectively establish that the major plant secondary metabolites identified in KP are present and unaltered from those found in TKT.

### 3.2 Participant selection criteria and demographic characteristics

Results in Table 3 summarize the demographic characteristics of the participants who met the inclusion criteria of completion (n = 41). Participants were 54.29% female, 40% male, and 5.71% non-binary/genderfluid/other. All participants were adults with 34.29% aged 25–34, 51.43% aged 35–44, and 14.3% aged 44 and older. The education level among participants was 8.57% with High School degree or equivalent, 8.57% with some college education, 11.43% with trade or technical degrees, 17.14% with an associate’s degree, 37.14% with a bachelor’s degree, 11.43% with a master’s degree, and 5.71% with a professional degree. Many participants had previous experience with other products like *Cannabis* (85.4%), CBD (78.0%), Vitamin B12 (61.0%), Alcoholic seltzers (58.5%), tobacco (58.5%), and other kratom products (36.6%) as reported in Table 4.

**TABLE 3:** General demographics.

Variable	N	Percentage (%)
*Gender*		
Female	19	54.3
Male	14	40.0
Non-binary/Other	2	5.7
*Age*		
25–34	8	22.9
35–44	19	54.2
45+	8	22.9
*Highest Education*		
High school equivalent	3	7.3
Some college	3	7.3
Trade/Technical	4	9.8
Associate’s degree	6	14.6
Bachelor’s degree	13	31.7
Master’s degree	4	9.8
Professional degree	2	4.9

**TABLE 4:** Previous use of kratom and other products.

Product	N	Percentage (%)
Cannabis (marijuana)	35	85.4%
Vitamin B12	25	61.0%
Alcoholic seltzers	24	58.5%
CBD	35	78.0%
Tobacco	24	58.5%
Kratom	15	36.6%

### 3.3 Improvements in focus and productivity

Although participants reported a range of consumption, to assess the activity of the KP aligned with the serving size recommendation, only responses from participants that reported 20 mg mitragynine equivalent dose were evaluated (n = 35 participants, n = 230 observed experiences).

**FIGURE 2:**
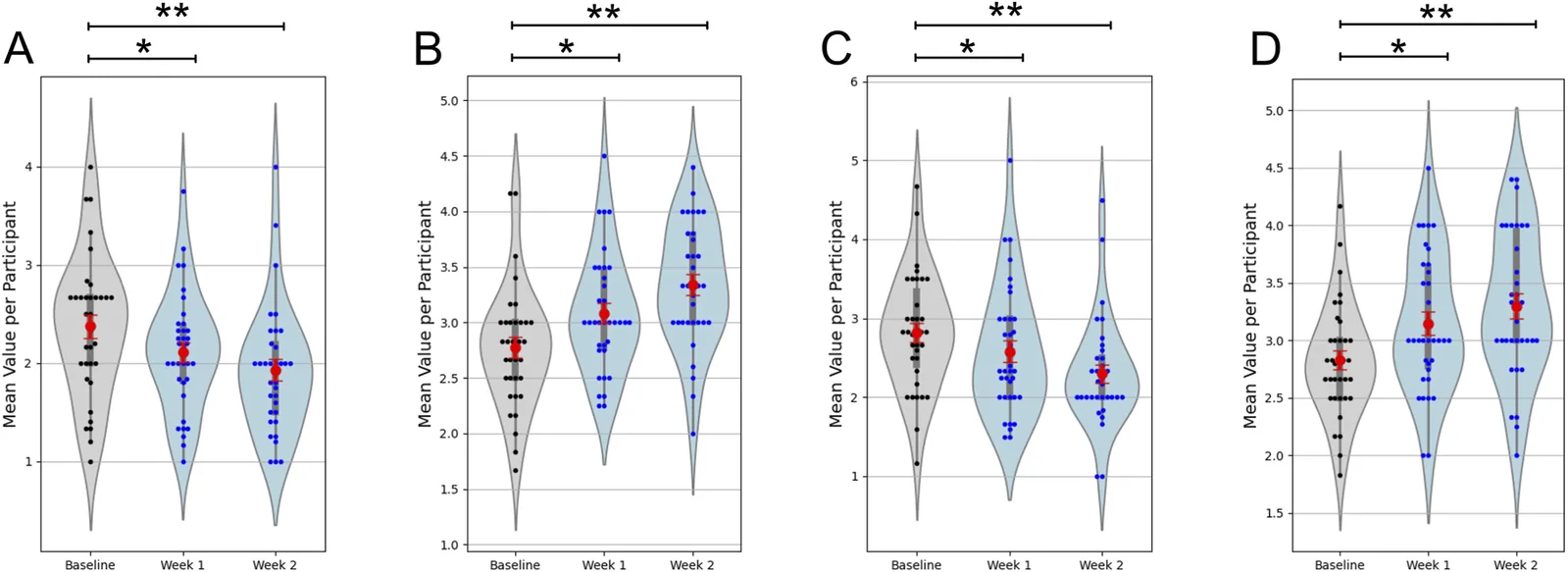
Self-reported improvements in focus and productivity scores of participants by week shown as individual datapoints over a violin distribution plot. **(A)** Thinking clearly (1 = Never … 5 = Always), **(B)** Focused on a single task (1 = Very Difficult … 5+Very easy), **(C)** Mind drift (1 = Never … 5 = Always), **(D)** Problem solving (1 = Very difficult … 5 = Very easy). (p*<0.05, **p < 0.001). Mean values and standard error are marked in red overlays for each phase.

There were significant improvements in self-reported outcomes for focus and productivity over the two-week observation period following initiation of KP. For the survey questions “How often did you have problems thinking clearly today?” (Figure 2A) and “How often did your mind drift away today when working on something?” (Figure 2C) participants responded on a Likert scale of 1 = Never, 2 = Sometimes, 3 = Regularly, 4 = Often, and 5 = Always, with a higher mean value score per participant indicating increased difficulty in thinking clearly and increased mind drift. For problems with thinking clearly, the mean baseline score was 2.38 ± 0.71, which decreased to 2.11 ± 0.60 in week 1 and 1.93 ± 0.66 in week 2, indicating that participants experienced a decrease in the frequency of problems with thinking clearly during the observational period (Figure 2A). This trend was also observed in frequency of mind drift when working on a task with the baseline mean of 2.82 ± 0.73 decreasing to 2.58 ± 0.81 in week 1 and 2.30 ± 0.69 in week 2.

For the questions “How easy or difficult was it for you to stay focused on one specific task today?” (Figure 2B) and “How easy or difficult was it for you today to focus your attention to solve a problem?” (Figure 2D) participants responded on a Likert scale of 1 = Very difficult, 2 = Difficult, 3 = Neutral, 4 = Easy, 5 = Very easy, with a higher mean value score per participant indicating increasing ease in focus and attention on a specific task or problem solving. For focus on a specific task, participants baseline ratings increased from an average of 2.78 ± 0.54 to 3.08 ± 0.54 in week 1 and 3.34 ± 0.55 in week 2 (Figure 2B). The progressive increase in participant means shows an increase in ability to focus on a specific task during the observational period from more difficult to neutral. A similar trend was seen with problem solving with an increase from the baseline mean of 2.83 ± 0.49 to 3.15 ± 0.60 in week 1 and 3.30 ± 0.63 in week 2 (Figure 2D), showing participants self-reported an increase in perceived ease at focusing attention for problem solving.

Because this study involved a secondary analysis of anonymized, real-world observational data, formal prospective scale development (e.g., item generation, cognitive interviewing, test–retest assessment) was not possible. To internally validate our question-set, we evaluated construct validity and internal consistency of the self-reported Likert items within the available dataset. For the two different effects categories - focus/productivity and energy levels - we calculated the Cronbach’s alpha (α) score for the questions used to evaluate participant responses for each concept evaluated. For focus/productivity, Cronbach’s alpha score was 0.87 indicating a good-fit for internal consistency over the four questions asked. Taken together, participants who reported using 20 mg of mitragynine equivalent KP experienced a general increase in ability and ease of focus and productivity during the observational period as compared to baseline. All four focus and productivity outcomes were found to be statistically significant improvements using linear mixed-effects modeling at this dose (Figure 2).

### 3.4 Perceived fatigue and increased energy levels

**FIGURE 3:**
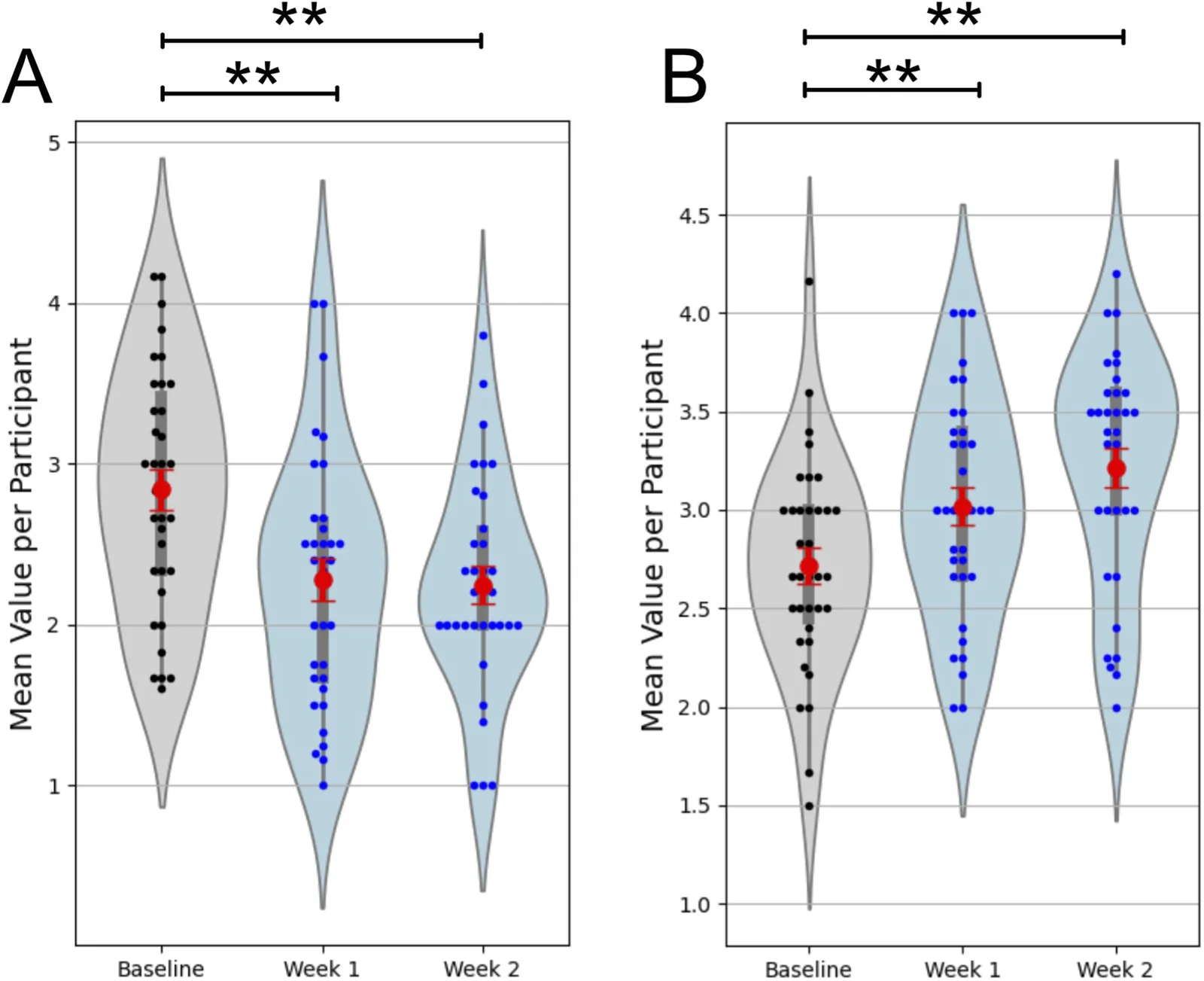
Self-reported increase in energy level scores per participant by week shown as individual datapoints over a violin distribution plot. **(A)** Feeling worn out (=Never … 5 = Always), **(B)** Overall energy level ratings (1 = No energy … 5 = Full of energy) (** = p < 0.001).

Self-reported energy levels and feelings of fatigue in the same participant and dose group (20 mg mitragynine equivalent KP) showed significant improvements over the observation period. Participants responded to the survey question “How much did you feel worn out today?” (Figure 3A) using a 5-point Likert scale of 1 = Never, 2 = Sometimes, 3 = Regularly, 4 = Often, and 5 = Always. There was a decrease in participants feeling fatigue from a baseline mean of 2.84 ± 0.75 to 2.28 ± 0.78 in week 1 and 2.25 ± 0.66 in week 2 (Figure 3A). Participant responses of feeling worn out more regularly throughout the day decreased to smaller windows of feeling fatigue. Participants also experienced changes in perceived energy levels. For the survey question “What was your energy level today?” participants responded using a 5-point Likert scale of 1 = No energy, 2 = Low energy, 3 = Moderate energy, 4 = Mostly energetic, and 5 = Full of energy. Participants reported a baseline mean of 2.72 ± 0.54, corresponding to low-moderate energy that increased to 3.02 ± 0.56 in week 1 and 3.21 ± 0.59 in week 2 (Figure 3B). Participants self-reported an increase in energy levels from the low-moderate range to moderate energy levels.

Linear mixed-effects modeling confirmed that both outcomes for fatigue and energy were statistically significant (p < 0.5) for participants in the 20 mg serving group. This data suggests that the 20 mg mitragynine dose has stimulatory properties that reduces fatigue and increases overall daily energy levels (Figure 3). For energy levels, Cronbach’s alpha score was 0.72 indicating an acceptable-fit for internal consistency for the two survey questions asked.

### 3.5 Perceived average daily pain, before and after product use

**FIGURE 4:**
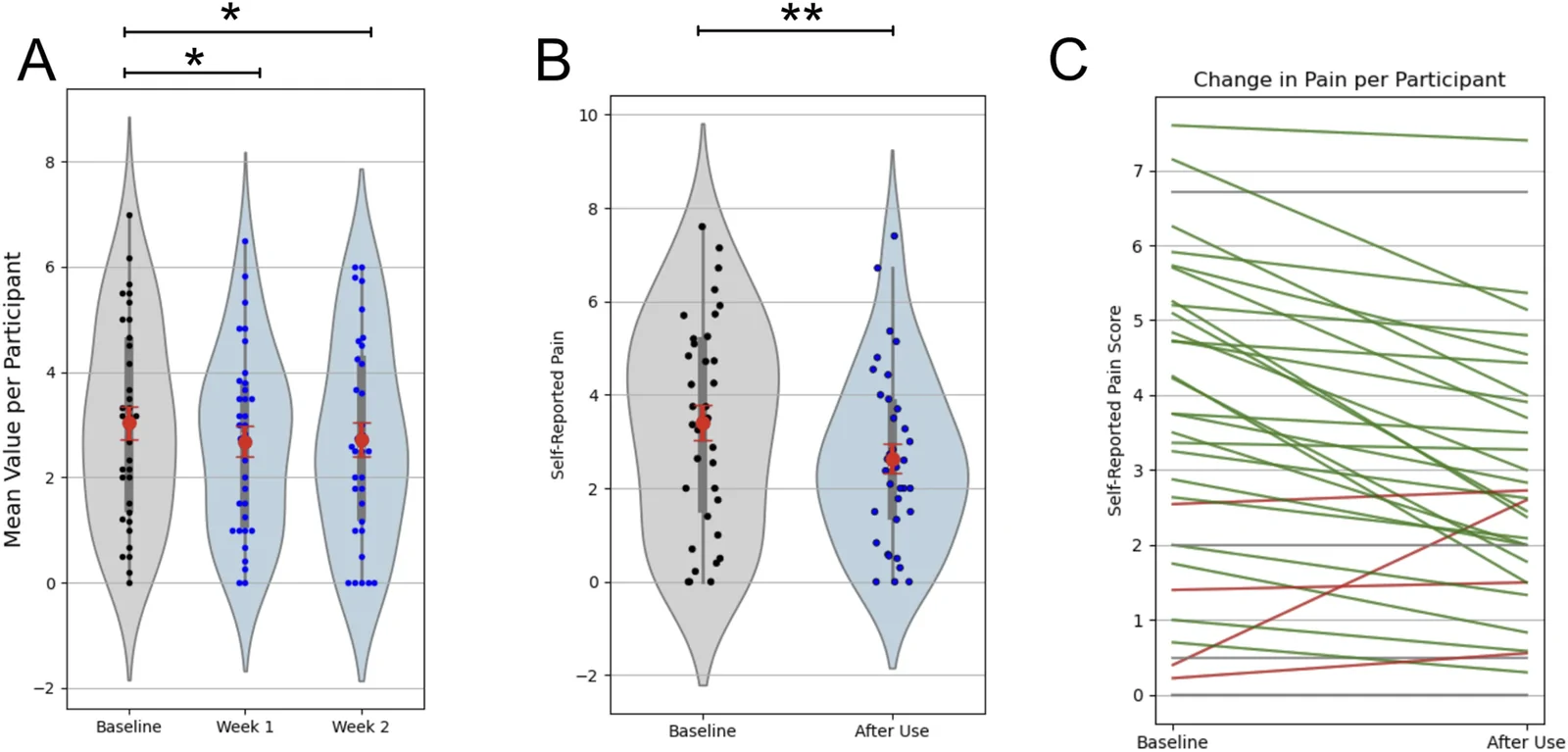
Self-reported decrease in pain scores per participant. **(A)** Self-reported average daily pain scores (0–10) per participant by week shown as individual datapoints over a violin distribution plot. **(B)** Self-reported pain before and after product use (0–10) shown as individual datapoints over a violin distribution plot and **(C)** shown as change per individual participant with participants experiencing a decrease in pain in green, no change in grey, and an increase in pain in red.

While less substantial than the changes reported in focus and energy levels, there was a significant perceived decrease in self-reported average pain levels from baseline to week 1 and week 2 of the observation period (Figure 4A) with a baseline mean of 3.04 ± 1.88, week 1 mean of 2.69 ± 1.711, and week 2 mean of 2.71 ± 1.89. On days where participants reported product use, they were asked to report their pain levels before and after product use. There was an observed decrease in perceived pain scores from an average of 3.40 ± 2.25 at baseline to an average of 2.64 ± 1.88 after product use (Figure 4B). This was statistically significant by paired t-test assessing individual participant’s responses, as visualized by Figure 4C.

### 3.6 Dose-specific profile and onset of additional side effects

**FIGURE 5:**
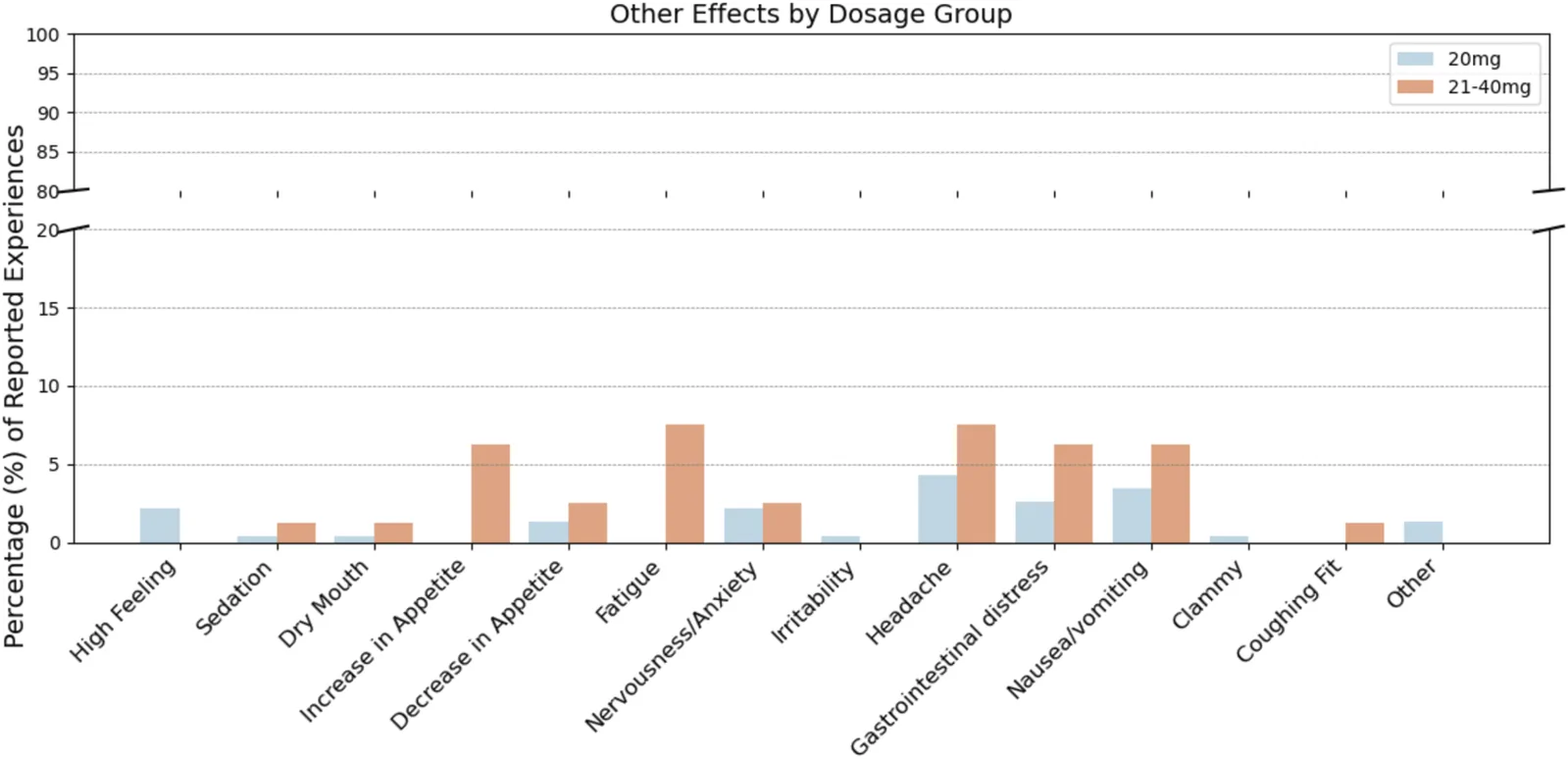
Distribution of percentage of total responses for all participants within each dose category for self-reported side effects reported by participants consuming a 20 mg mitragynine equivalent dose of KP (blue) and 21–40 mitragynine equivalent dose of KP.

Despite the serving recommendation of 20 mg mitragynine equivalent, participants self-reported a range of different doses throughout the observed period. Data from two different dose categories was filtered based on self-reported amounts. Analysis of the two dose categories, 20 mg and 21–40 mg, showed a significant difference in the overall distribution of effects experienced by percentage of reported experiences in each category. A total of n = 230 experiences were reported in the 20 mg mitragynine equivalent category and n = 80 reported experiences in the 21–40 mg mitragynine equivalent category. For the 20 mg mitragynine equivalent group, negative effects were reported in 14.8% of sessions (Table 5), with the most common side effect of headaches occurring in less than 5% of reported sessions. In contrast the 21–40 mg equivalent dose group saw negative reported effects rise to 23.8% of sessions with significant increases in the percentage of reported negative effects experienced. In particular, participants reported fatigue and greater changes in appetite, suggesting that there is an onset of new and differential effects at higher exposures and that increasing the total dose increases the likelihood and diversity of side effects experienced (Figure 5).

**TABLE 5:** Reports of negative effects.

Dosage group	N	Percentage (%)
20 mg (n = 230)	34	14.8%
21–40 mg (n = 80)	19	23.8%

## 4 Discussion

Today, kratom use is common in Southeast Asia, with an estimated >8 million consumers in Thailand alone (Tanguay, 2011; Wonguppa and Kanato, 2017), and in North America with an estimated 16 million American consumers (Henningfield et al., 2022). Although much remains unknown, available data, including an extensive history of use, human clinical studies, and animal toxicology studies are showing that traditional kratom has a low risk profile with respect to acute and chronic uses (Assanangkornchai et al., 2007; Singh et al., 2014; Swogger et al., 2015; Grundmann et al., 2018; Garcia-Romeu et al., 2020; Davidson et al., 2021; Henningfield et al., 2021; Smith et al., 2022b; Grundmann et al., 2023b; Huestis et al., 2026). However, the increase in popularity of kratom has resulted in a variety of available products, with a myriad of potencies and recommended serving sizes (Grundmann et al., 2023a; Grundmann et al., 2023b).

It is important to differentiate between traditional preparations and non-traditional formulations as dosing, bioavailability and pharmacokinetic profiles of active constituents can be altered such that resulting products are no longer chemically, functionally or biologically related to traditional kratom (Grundmann et al., 2023a; Smith et al., 2024a; Brown et al., 2026; Gour et al., 2025). The history of safe and beneficial use is not generally applicable to all kratom products in the modern market, but rather, apply only to those products prepared as per tradition and consumed at typical serving sizes (Tanguay, 2011; Singh et al., 2019a; Singh et al., 2019b). As such, any novel or non-traditional product should be independently evaluated for safety and efficacy.

In this analysis the effects of KP, formulated with a kratom leaf extract and standardized to mimic a traditional kratom tea serving consistent with traditional consumption patterns in Southeast Asia, was investigated. By establishing phytochemical equivalence between the KP and traditionally prepared kratom through analytical comparisons we have created a basis for investigating traditional use outcomes for a modern, standardized product. Although chemical characterization establishes phytochemical similarities of KP with products used in traditional practice, KP is still a modern product that is unique from other products and traditional preparations. As such, studies evaluating its specific effects are warranted.

While well designed clinical studies would be the most informative, real-world data (RWD), collected under everyday conditions and outside the constraints of clinical settings, fills the data gap between qualitative traditional knowledge and how today’s consumers use natural products. This real-world observational study provides preliminary evidence that consumption of a KP formulated in alignment with ethnopharmacological use results in improvements in focus, problem-solving, mental clarity, and overall energy levels. These findings are consistent with traditional reports of kratom use as a restorative with stimulant enhancing properties and support studies suggesting additional non-opioid mechanisms at lower doses, potentially through the adrenergic, dopaminergic, and serotoninergic systems (Matsumoto et al., 1996; Hazim et al., 2014; Obeng et al., 2020; Stolt et al., 2014; León et al., 2021).

While there are gaps in understanding the dose-response effects for traditional kratom preparations, in general the stimulating and focus-enhancing effects is associated with low to moderate doses of 1–5 g of leaves (Prozialeck et al., 2012; Grundmann, 2017; Smith et al., 2022b). In contrast, higher doses have been reported to have sedative effects, along with a higher risk and incidence of adverse effects (Prozialeck et al., 2012; Grundmann, 2017; Smith et al., 2022b). This suggests there are separate therapeutic windows for the different pharmacological effects of kratom. The self-reported decrease in average pain and perceived pain before and after product use at the 20 mg equivalent dose suggests that even at doses relevant as compared to traditional preparations, kratom exhibits some analgesic effects. The specific threshold between what constitutes a low and a high dose is not clear, however the use of ethnobotanical knowledge can guide towards traditional doses that were used to achieve desired effects.

The adverse effects experienced at the recommended KP serving of 20 mg mitragynine equivalent were relatively infrequent with fewer than 15% of total sessions reporting negative effects. The most common side effect, headache, was reported in less than 5% of reported sessions. Reports from the higher consumption group (<20–40 mg mitragynine equivalent) showed an increase in prevalence of side effects and shift in the overall profile with 24% of reported side effects representing onset of fatigue, sedation, and changes in appetite. These findings are consistent with the known dose-dependent pharmacology of mitragynine, whereby higher doses appear to increase activity at the opioid system, producing effects like fatigue and sedation.

With any substance utilized with the end-goal of increasing focus and energy, including caffeine, nicotine, and amphetamines, consumers often must self-determine the net benefit of these effects. The self-reported improvements in focus and energy at the 20 mg mitragynine equivalent dose are modest in magnitude. For regular servings, as described in the American Herbal Product Association Botanical Safety Handbook, adverse effects are rare and relatively minor (Assanangkornchai et al., 2007; Coe et al., 2019; Davidson et al., 2021; Garcia-Romeu et al., 2020; Grundmann, 2017; Grundmann et al., 2023c; Henningfield et al., 2021; Singh et al., 2014; Smith and Lawson, 2017; Smith et al., 2022b; Swogger et al., 2015). A regular serving is described as 1–5 g of kratom leaves (Grundmann et al., 2023c) containing from 2.1 to 24.0 mg/g mitragynine (Lydecker et al., 2016; Mudge and Brown, 2018; Parthasarathy et al., 2010; Sengnon et al., 2023) for a range of 2.1–120.0 mg of mitragynine. Kratom dependency and withdrawal symptoms are dose and duration dependent with symptoms rarely reported among users consuming regular doses as described above (Garcia-Romeu et al., 2020; Grundmann, 2017; Singh et al., 2014). The few studies that report on occasional kratom use rarely report dependency and withdrawal symptoms, and for those rare cases where adverse events are reported, the effects were considered to be relatively benign, of short duration and well tolerated, not unlike caffeine (Garcia-Romeu et al., 2020; Grundmann, 2017; Singh et al., 2014; Rodak et al., 2021).

While this analysis provides valuable real-world insights on the use of a kratom product, several limitations should be acknowledged. First, it relied upon a relatively small sample size of self-reported observational data to reach full completion over 21 days, especially for the number of sessions reported for the 21–40 mg dose equivalent group which limits statistical power and generalizability. The self-selected dosage and self-reported data may have inconsistent dosing patterns between participants that may lead to more variable outcomes. Participants were recruited from a convenience sampling strategy of individuals engaged in health and wellness, introducing the potential for self-selection bias and limiting the applicability to the broader kratom-using population. Additionally, outcomes were based on self-reported measures collected under real-world conditions rather than clinical or cognitive assessments. Although this strategy makes data collection more accessible, the results may be influenced by recall bias or an expectancy effect. The observational period captured only 2 weeks of KP use, which cannot account for tolerance and other effects of chronic or extended use. Lastly, the observational design of this study does not account for potential confounding variables such as sleep quality, caffeine intake, other stimulant use, diet, stress levels, workload, and use of other bioactive substances; all of these have the potential to influence outcomes of focus, energy and mental clarity. Future studies with longer follow-up data collection periods are needed to further characterize these dynamics. Despite these limitations, real-world observational data plays an integral role in bridging traditional knowledge and controlled clinical research. These findings should be interpreted as exploratory, informing future controlled studies rather than establishing clinical efficacy.

## 5 Conclusion

This study represents an important bridge between ethnopharmacological knowledge on a traditionally used natural product and modern observational data. Real-world evidence supports that consumption of a full-spectrum kratom extract at doses aligned with ethnopharmacological use (20 mg mitragynine equivalent) enhances focus, problem solving, mental clarity, and energy with a favorable side-effect profile. Higher doses (21–40 mg) were associated with increased frequency and diversity of negative effects, highlighting the importance of dose and adherence serving size recommendations in order to balance benefits and tolerability. These findings support the use of a full spectrum kratom extract at doses comparable to traditional preparations, to enhance focus and energy with a tolerable side effects profile. This study also highlights the need for more investigation into the chemical diversity of plant-based natural medicines, which involve complex interactions among multiple bioactive compound classes. By studying natural formulations rather than isolated synthetic analogues, researchers can better capture the nuanced pharmacology that has supported traditional use of these natural products for centuries.

## Data Availability

The raw data supporting the conclusions of this article will be made available by the authors, without undue reservation.
